# Different Reconstitution Behaviors of Two Poly‐L‐Lactic Acid Biostimulator Formulations: A Mechanistic Hypothesis

**DOI:** 10.1111/jocd.71081

**Published:** 2026-08-07

**Authors:** Chuan‐Yuan Lin, QianZhong Zhang, Jui‐Yu Lin, Qiong Meng

**Affiliations:** ^1^ Nanzi Annyeong Clinic Kaohsiung Taiwan; ^2^ Chengdu Royalty Aesthetic Clinic Chengdu China; ^3^ Hangzhou Royalty Aesthetic Clinic Hangzhou China; ^4^ School of Pharmaceutical Sciences Sun Yat‐Sen University Guangzhou China; ^5^ Meiyan Space, Sihuan Pharmaceutical Holdings Group Ltd. Beijing China; ^6^ Hunan University of Medicine Huaihua China

**Keywords:** carboxymethylcellulose, degree of polymerization, degree of substitution, lyophilized biostimulator, reconstitution

To the Editor,

Poly‐L‐lactic acid (PLLA)–based biostimulators are increasingly used in the aesthetic field. Lyophilized formulations containing carboxymethyl cellulose (CMC) must be reconstituted with sterile water for injection to form a homogeneous suspension prior to administration, and the dissolution behavior of CMC can influence the gross appearance and handling characteristics of the resulting suspension [1, 2]. In our repeated routine reconstitution observations involving more than 1000 vials of each formulation, PLLA‐SCA (Sculptra; Galderma Laboratories) and PLLA‐SIF (Sifuyan; Meiyan Space, Sihuan Pharm, Beijing, China) consistently appeared to show different gross reconstitution behaviors despite having the same labeled amounts of PLLA, CMC, and mannitol per vial. Here, we compare these observed reconstitution behaviors and discuss one possible physicochemical explanation.

A vial of PLLA‐SCA contains 150 mg of PLLA microparticles, 90 mg of CMC, and 127.5 mg of mannitol. Upon reconstitution, the CMC dissolves to form a viscous solution that suspends the water‐insoluble PLLA microparticles. However, CMC has an inherent tendency to form lumps when hydrated. Pre‐mixing CMC with mannitol helps distribute CMC molecules and reduce large‐lump formation, yet small micro‐lumps—often termed “CMC micro‐fisheyes,” consisting of partially hydrated particles with dry cores—may still develop. These micro‐lumps tend to float on the suspension surface and may be relevant to needle clogging; they have also been discussed as a possible contributing factor to delayed‐onset nodules or papules following injection [2, 3]. Prolonging hydration time reduces micro‐lump formation but does not reliably eliminate it [1, 2, 3] (Figure 1A). Historically, longer hydration times were recommended to reduce this problem, although more recent analytical and clinical studies suggest that immediate use of PLLA‐SCA can also be feasible under specific reconstitution protocols [4].

**FIGURE 1:**
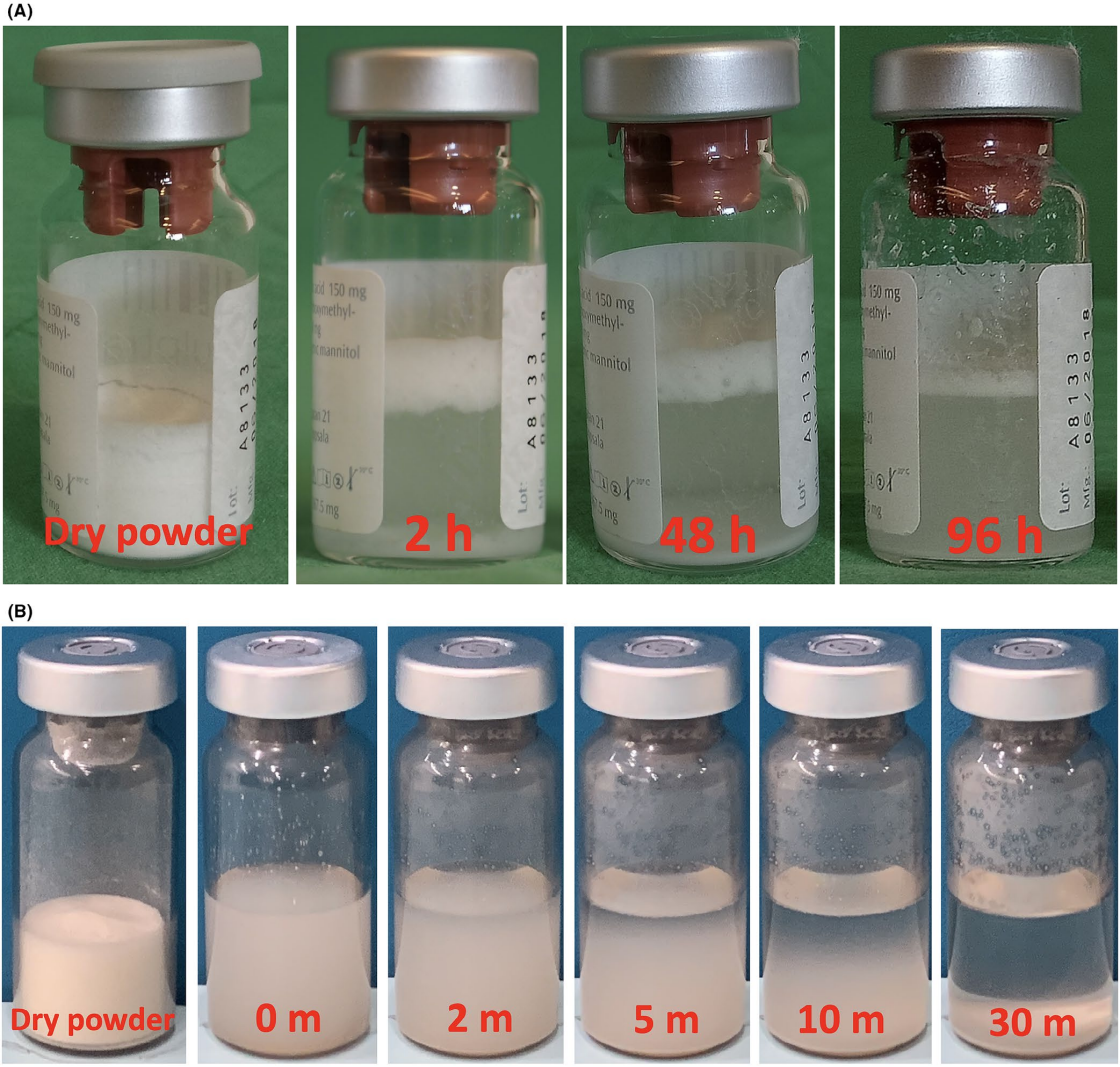
Representative gross observations during reconstitution of 2 PLLA‐based biostimulator formulations with 5 mL of sterile water for injection. (A) Representative gross observations during reconstitution of PLLA‐SCA after addition of sterile water for injection and shaking for 5 min. The image panels from left to right show: dry powder, 2, 48, and 96 h after reconstitution. No grossly visible CMC lump formation was observed. A floating upper layer, interpreted as CMC micro‐lumps, gradually decreased with prolonged hydration time (Lin et al. [1]; with permission). (B) Representative gross observations during reconstitution of PLLA‐SIF after addition of sterile water for injection and shaking for 1 min. The image panels from left to right show: dry powder, 0, 2, 5, 10, and 30 min after reconstitution. No grossly visible CMC lumps or micro‐lumps were observed during the early phase after reconstitution. The suspension remained grossly homogeneous for approximately the first 2–3 min, after which gradual particle sedimentation became apparent. The appearance became relatively stable by approximately 30 min after reconstitution. Because the 2 formulations exhibited different temporal patterns of reconstitution behavior during routine preparation, the observation intervals shown in (A,B) were selected to illustrate representative phases of each process rather than to establish synchronized experimental comparison conditions.

A vial of PLLA‐SIF also contains 150 mg of PLLA microparticles, 90 mg of CMC, and 127.5 mg of mannitol. In our observations, the suspension became grossly homogeneous almost immediately upon reconstitution and showed no obvious CMC micro‐fisheyes. However, the suspension remained homogeneous for only 2–3 min after shaking, after which gradual particle sedimentation became apparent (Figure 1B). One possible explanation is that differences in CMC‐related structural parameters may contribute to the distinct reconstitution behaviors observed in these two formulations.

CMC is produced by substituting hydroxyl groups on the cellulose backbone with carboxymethyl groups, thereby conferring water solubility. In general, a higher degree of substitution (DS) and greater substitution uniformity are expected to enhance solubility. The degree of polymerization (DP) also influences solubility: a higher DP yields longer polymer chains with greater chain entanglement and intermolecular interactions, making hydration and chain separation more difficult [5, 6]. On this basis, differences in CMC structural parameters could plausibly contribute to the distinct reconstitution behaviors observed here, although this remains only one possible explanation.

If the CMC in PLLA‐SIF yields a lower‐viscosity aqueous phase after dissolution, faster post‐shaking microparticle precipitation could also be expected on theoretical grounds. Consistent with this possibility, in our observation the suspension remained grossly homogeneous for approximately 2–3 min after shaking, after which gradual precipitation became apparent. This behavior may have practical implications for handling. Because visible sedimentation was observed within a few minutes after shaking, there may be a theoretical concern regarding dose uniformity during clinical use if the suspension is allowed to stand. Earlier syringes or aliquots could theoretically contain fewer suspended microparticles, whereas later injections might contain relatively more concentrated suspensions. Such variability could potentially influence treatment reproducibility, local microparticle distribution, inflammatory response, and possibly clinical efficacy.

This report has several limitations. No direct experimental data were available regarding the physicochemical properties of the CMC used in either product. Quantitative viscosity testing and sedimentation analysis were not performed. In addition, the CMC‐related explanation proposed here should be regarded as only one possible working hypothesis rather than a demonstrated mechanism. Variables other than CMC, including PLLA microparticle size distribution, microparticle morphology, agglomeration behavior, lyophilization architecture, residual moisture content, and manufacturing process variables, may also influence hydration behavior, suspension stability, and sedimentation kinetics. Similarly, the possible effects of the rapid sedimentation observed in PLLA‐SIF described above remain theoretical and require formal evaluation. The present comparison should therefore be regarded as an exploratory, hypothesis‐generating observation rather than evidence of clinical superiority or inferiority of either formulation.

In conclusion, PLLA‐SCA and PLLA‐SIF appear to exhibit different gross reconstitution behaviors despite having the same nominal component amounts per vial. This observation suggests that the handling characteristics of PLLA‐based biostimulators may be formulation‐specific and should not be inferred solely from labeled component composition. Future standardized studies are needed to clarify the practical significance of these observations.

## Ethics Statement

The authors have nothing to report.

## Consent

The authors have nothing to report.

## Conflicts of Interest

Q.Z. is the product manager of Meiyan Space. Q.M. is the medical director of Meiyan Space. C.‐Y.L. and J.‐Y.L. have no conflicts of interest to disclose.

## Data Availability

Data sharing not applicable to this article as no datasets were generated or analysed during the current study.
